# Perceived usefulness of online videos for hereditary breast cancer communication in Japan: Patient and provider perspectives

**DOI:** 10.1016/j.puhip.2026.100845

**Published:** 2026-08-22

**Authors:** Asumi Iesato, Hitomi Suzuki, Rie Tahara, Mitsuo Terada, Yuko Nakazawa, Madoka Iwase, Tomoko Shibayama, Chihiro Kawamura, Nami Yamashita, Atsushi Fushimi

**Affiliations:** a Cancer Cell Diversity Project, NEXT-Ganken Program, Japanese Foundation for Cancer Research, Tokyo, Japan; b General Incorporated Association BC TUBE, Tokyo, Japan; c General Incorporated Association Peer Ring, Kanagawa, Japan; d Department of Breast Surgery, Nagoya City University Graduate School of Medical Sciences, Nagoya, Japan; e Department of General Surgical Science, Gunma University Graduate School of Medicine, Gunma, Japan; f Department of Breast and Endocrine Surgery, Nagoya University Hospital, Aichi, Japan; g Machida Ekimae Breast Clinic, Tokyo, Japan; h Breast Oncology Center, Cancer Institute Hospital of Japanese Foundation for Cancer Research, Tokyo, Japan; i Department of Surgery, The Jikei University School of Medicine, Tokyo, Japan

**Keywords:** Hereditary breast cancer, Genetic information, Patient survey, Healthcare provider survey, Online video, Patient education

## Abstract

**Objectives:**

Evidence-based online videos may supplement hereditary breast cancer (BC) communication, but their perceived usefulness in Japan is unclear. We assessed information sources, perceived comprehension, family communication intentions, and stated willingness to use online videos among women in an online cancer peer-support community and healthcare providers.

**Study design:**

Cross-sectional online survey.

**Methods:**

We surveyed female patients in a Japanese online peer-support community for women with breast, cervical, or ovarian cancers (January–February 2024) and healthcare providers recruited at a national BC meeting (June–July 2023). Items addressed information sources, perceived understanding of BC genetics, shared treatment decision-making, family communication intentions, preferred educational formats, and willingness to use evidence-based online videos. Analyses were descriptive, and item-specific denominators were used.

**Results:**

Among 375 patient respondents, 88.7% (251/283) reported understanding of BC genetics, whereas 28.1% (64/228) reported difficulty understanding some aspects. Physicians and medical institutions were the most trusted information source, although internet use was common. Among respondents to the decision-making item, 51.8% (118/228) reported being able to make their own treatment decisions and 30.7% (70/228) reported being able to do so to some extent. Most respondents intended to share genetic information with adult family members (72.5%, 272/375) and, where applicable, adolescent family members (65.5%, 74/113). Among providers, 20.0% (6/30) felt able to convey all necessary information in routine practice, while 86.7% (26/30) were interested in using online video resources. Providers prioritized clarity, evidence-based content, and text-and-illustration materials.

**Conclusions:**

In two selected samples, patients and providers supported evidence-based online videos as an adjunct to hereditary BC communication. Findings reflect self-selected respondents already engaged with digital health information or educational resources and should not be generalized to broader Japanese patient or provider populations. Prospective studies using validated measures are needed to evaluate implementation strategies and effects on knowledge, decisional conflict, anxiety, and family communication.

## Highlights

• First Japanese study of patient and provider views on online videos for hereditary breast cancer communication.
• Patients trusted physicians and institutions but also commonly used online sources; some reported comprehension difficulties.
• Patients and providers supported online videos, but perceived usefulness was assessed without a defined video intervention.
• Evidence-based online videos may supplement hereditary breast cancer communication and allow patients to revisit information.
• Future resources should be trusted, age-appropriate, family-centered, and prospectively evaluated using validated outcomes.

## 1 Introduction

Communication of hereditary breast cancer (BC) risk is inherently complex and cognitively demanding. Patients considering *BRCA1/2* genetic testing, risk-reducing surgery, or intensive surveillance must integrate probabilistic genetic concepts, personal values, and considerations for at-risk relatives, typically within time-limited clinical encounters. Gaps in understanding can undermine informed decision-making, adherence to risk-management options, and effective family communication.

Pathogenic germline variants in *BRCA1* and *BRCA2* define hereditary breast and ovarian cancer (HBOC) and confer substantially elevated lifetime risks of breast and ovarian cancers. [1] In Japan, insurance coverage for *BRCA1/2* testing and related risk-management options has expanded since 2020, and the Japanese Organization of HBOC has established a nationwide framework for designated facilities. [[2], [3], [4], [5]] As access to genetic services and preventive care expands, ensuring that patients and their families can meaningfully understand, discuss, and act on hereditary cancer information has become an increasingly important public-health priority.

Internationally, structured genetic counseling and formal decision aids, including web-based formats, can improve knowledge and reduce decisional conflict among individuals considering *BRCA1/2* testing and risk-reducing options. [[6], [7], [8], [9]] However, limited consultation time may constrain opportunities to ensure comprehension and support family communication. Patients also frequently access medical information online, where quality is variable. [[10], [11], [12], [13]] Beyond individual counseling, international studies have shown variable intra-family disclosure of *BRCA1/2* risk, often mediated by probands, and persistent challenges in communication with children and adolescents. [14,15] In Japan, BC TUBE, a nonprofit educational YouTube channel, provides animated, evidence-based videos on BC, including hereditary BC; its videos have been rated higher in scientific validity and clarity than other online media, although lower-quality content can attract greater attention. [16,17]

Despite the growing availability of evidence-based online video resources in Japan and internationally, patient and healthcare-provider perspectives on their perceived usefulness for hereditary BC communication in Japan remain unclear. Little is known about how patients evaluate online video resources in relation to their information needs and family communication practices, or how healthcare providers view such resources in relation to their day-to-day workflows. This limits the design and integration of scalable, evidence-based educational materials aligned with the needs of both groups.

To address this gap, we conducted a cross-sectional survey of women in an online Japanese cancer peer-support community for breast, cervical, and ovarian cancers, together with healthcare providers attending a national BC meeting. Our objectives were to describe patterns of information seeking and provision and to evaluate perceived usefulness and stated willingness to use evidence-based online video resources. Because participants were not exposed to a specific video intervention, our findings reflect attitudes toward such resources rather than demonstrated acceptability or effectiveness of an implemented tool.

## 2 Methods

### 2.1 Survey of female patients with cancer

**Eligibility criteria.** All registered members of the online peer-support community “Peer Ring” were eligible to participate. Peer Ring is a Japan-based online platform serving women diagnosed with breast, cervical, or ovarian cancers; under the supervision of medical advisors, it provides an online environment where members can connect and exchange information and experiences with others who have similar diagnoses. Inclusion required current active membership of Peer Ring at the time of invitation. There were no exclusion criteria based on age, region, time since diagnosis, treatment status, or genetic-testing status. Participation was not restricted by cancer type, and no item asked respondents to specify their individual diagnosis or genetic-testing status.

**Source population and rationale.** Peer Ring was selected because it is a large Japanese online peer-support community for women with breast, ovarian, and cervical cancers; the study focused on information needs and educational resources from the perspective of cancer-affected women; and recruitment from a hospital-based HBOC cohort would have produced an even more selected sample of women already enrolled in formal genetic counseling. As of March 2024, Peer Ring had 17,076 registered members.

Eligible participants received an invitation email from the Peer Ring operations team in January 2024 that outlined the research objectives, contact information for inquiries, confidentiality measures, and the voluntary nature of participation. A single reminder email was sent approximately three weeks after the initial invitation; the survey was kept open for the entire planned January–February 2024 window regardless of the running response total.

**Sample size.** As this was an exploratory descriptive study, no formal a priori sample-size calculation was performed. We aimed for a response total similar to a prior internal Peer Ring survey, which obtained 360 responses.

**Questionnaire and instrument development.** The questionnaire was developed by the BC TUBE editorial committee and Peer Ring operations team, based on a prior internal survey and Japanese HBOC educational materials. Items were iteratively reviewed within the editorial committee, but no formal cognitive interviewing, pilot pretesting, or psychometric validation was performed. The Japanese questionnaire contained 32 items and is provided with an English translation in Supplementary Table 1. The questionnaire included items on family communication, including sharing information with adolescent family members. Several terms, including “search for information”, “materials received from hospitals”, and “shareable information”, were not pre-defined for respondents. The treatment decision-making item was interpreted as perceived participation in shared treatment decision-making.

The questionnaire was administered via Google Forms. The first page explained the study purpose, confidentiality, voluntary participation, and that survey completion indicated consent. Ethical approval was obtained from the Ethical Review Board of Ichinomiya Nishi Hospital (Approval No. 2022002).

### 2.2 Questionnaire survey of healthcare providers

**Eligibility criteria and recruitment context.** Eligible providers were healthcare professionals involved in BC care, including oncologists, breast surgeons, certified genetic counselors, clinical geneticists, oncology nurses, breast ultrasound technologists, and rehabilitation-focused physical therapists. Eligibility was confirmed by brief oral screening. The survey was conducted at the BC TUBE booth in the official exhibition area of the 2023 Japanese Breast Cancer Society Annual Meeting. The booth was hosted by BC TUBE to introduce its educational video platform, and survey recruitment was one component of the booth activity. The total number of eligible booth visitors was not enumerated, so a response rate could not be calculated.

In Japanese clinical practice, hereditary BC information is typically discussed by certified genetic counselors, clinical geneticists, and breast surgeons or medical oncologists involved in HBOC care, with oncology nurses often contributing in a supportive role. Breast ultrasound technologists and rehabilitation-focused physical therapists were included because they have direct patient contact across the BC care pathway, although they are not typically the primary providers of genetics-related counseling.

**Facility classification.** “HBOC core facility” and “HBOC cooperative facility” are JOHBOC designations. Core facilities provide the full HBOC care pathway, whereas cooperative facilities provide selected services in collaboration with a core facility. “Other/none of the above” refers to facilities outside this designated network.

**Provider questionnaire.** The provider questionnaire was developed by the BC TUBE editorial committee using the same source materials and iterative review process. No formal cognitive interviewing, pilot pretesting, or psychometric validation was performed. The Japanese questionnaire contained 27 items and is provided with an English translation in Supplementary Table 2. The questionnaire did not pre-define “shareable information” or occupational role labels; for analysis, “medical laboratory technician” was grouped as breast ultrasound technologist, and “shareable information” as patient-facing materials intended for onward sharing with family members. The survey also included an open-ended item on topics or contexts difficult to explain about BC and genetics. By responding, participants consented to participate. Thirty providers responded to the provider survey items analyzed in this study.

### 2.3 Data presentation

All analyses were descriptive. We computed frequencies and proportions for categorical items using item-specific denominators based on the relevant survey item and skip pattern. Both surveys allowed item non-response, and we did not impute missing values. Item-specific denominators are reported throughout the results, figure legends, and table footnotes; an item-by-item summary of denominators, responses excluded from denominators, and explicit “not specified” responses included in denominators is provided as Supplementary Table 3. “Not specified” in the figures and tables corresponds to respondents who selected an explicit “not specified/prefer not to say” response option on a given item, and is distinct from item-level missing responses.

Free-text provider responses to the open-ended item on topics or contexts difficult to explain were analyzed using a simple inductive content-analysis approach. The first author (a physician) coded the responses; an initial code list was developed from a first pass through all responses, refined through a second pass, and reviewed by a second author with experience in cancer communication research. Disagreements were resolved by discussion and re-reading of the original Japanese-language responses. No formal coding guide and no inter-rater reliability statistic was generated, given the small number of responses; the resulting categories are summarized here as simple counts. No inferential statistical tests were performed.

R software was used for data handling, and GraphPad Prism 8 (GraphPad Software, San Diego, CA) was used for figure generation.

## 3 Results

### 3.1 Patient survey

#### 3.1.1 Respondent characteristics

Of the 17,076 invited Peer Ring members, 375 completed the patient survey (response rate: approximately 2.2%). Participants were categorised by genetic-counseling status and information-seeking behavior into three primary strata: no genetic counseling/no genetic-information search (NC/NS) (n = 93, 24.8%), no genetic counseling/genetic-information search (NC/S) (n = 193, 51.5%), and genetic counseling/genetic-information search (C/S) (n = 87, 23.2%) (Table 1). The genetic counseling/no genetic-information search (C/NS) stratum (n = 2) is not displayed in Table 1 owing to small cell size but is included in overall analyses. Regional analysis showed the highest number of responses from Kanto (Tokyo metropolitan area), with substantial contributions also from Kinki (Osaka–Kyoto area) and Chubu. Demographic distributions across strata (age, partner status, presence of siblings and children) are summarized in Table 1.

**Table 1:** Characteristics of the respondents from the women's cancer patient group.

N	375	NC/NS	NC/S	C/S
		93 (24.8)	193 (51.5)	87 (23.2)
Region	Kanto	56 (60.3)	103 (53.4)	38 (43.6)
	Chubu	13 (14.0)	34 (17.6)	23 (26.4)
	Kinki	10 (10.8)	26 (13.5)	19 (21.8)
	Overseas	1 (1.1)	0 (0)	0 (0)
	Other	13 (14.0)	30 (15.5)	7 (8.0)
Age	<40-	1 (1.1)	10 (5.2)	5 (5.7)
	40-	19 (20.4)	68 (35.2)	30 (34.5)
	50-	45 (48.4)	82 (42.5)	39 (44.8)
	60-	25 (26.9)	30 (15.5)	13 (14.9)
	70-	3 (3.2)	3 (1.6)	0 (0)
Partner, spouse	No	21 (22.6)	46 (23.8)	18 (20.7)
	Yes	72 (77.4)	145 (75.1)	68 (78.2)
	Other	0 (0)	2 (1.0)	1 (1.1)
Siblings	No	10 (10.8)	10 (5.2)	9 (10.3)
	Yes	80 (86.0)	179 (92.7)	77 (88.5)
	Other	3 (3.3)	4 (2.1)	1 (1.1)
Children	No	41 (44.1)	65 (33.7)	23 (26.4)
	Yes	52 (55.9)	126 (65.3)	64 (73.6)
	Other	0 (0)	2 (1.0)	0 (0)

#### 3.1.2 Understanding and sources of information on BC genetics among the survey respondents

**Fig. 1:**
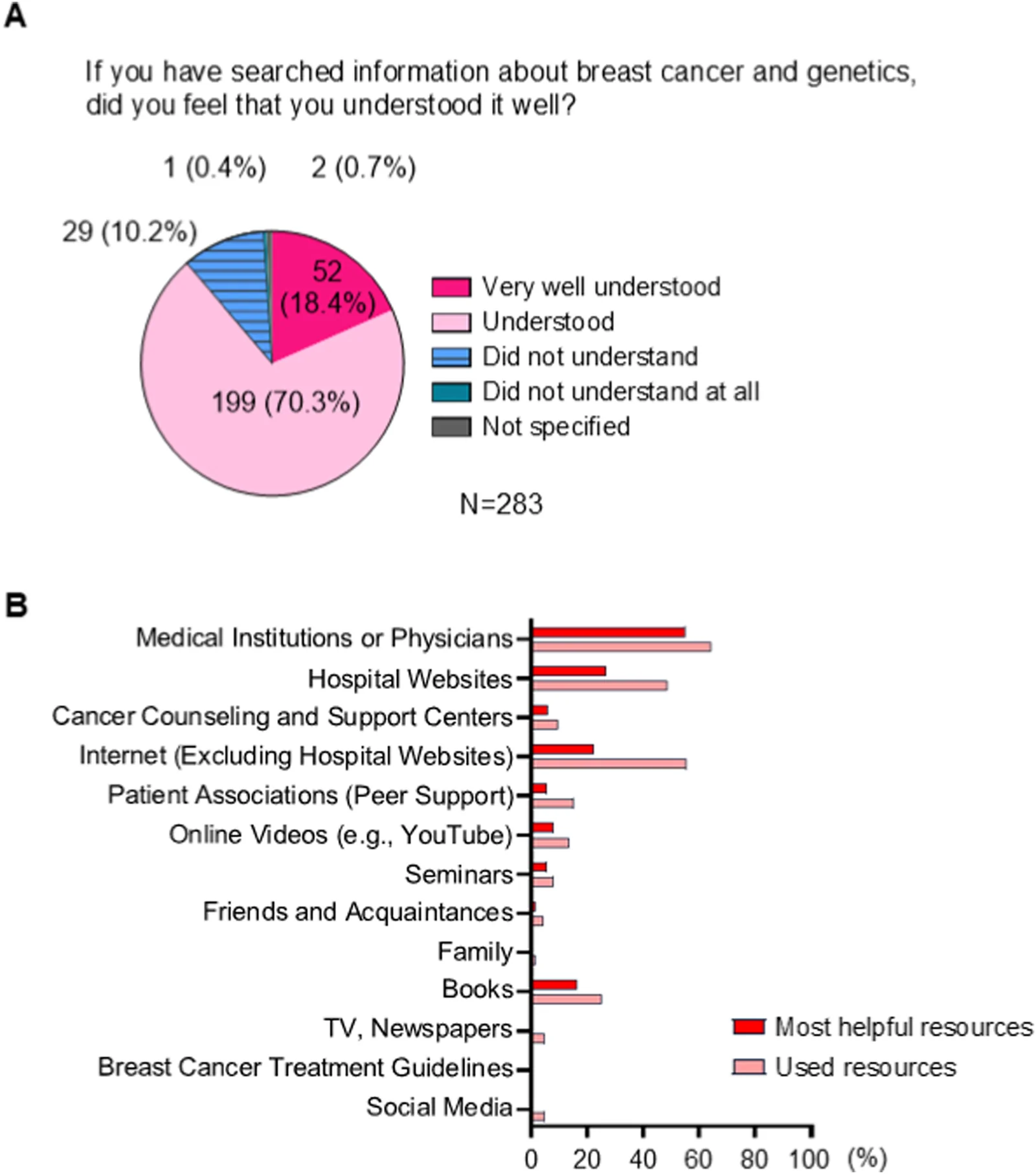
**Patient understanding and information sources about breast cancer genetics**. (A) Understanding among respondents who had searched for breast cancer genetics information (n = 283; explicit not-specified responses included). (B) Sources used and perceived as most helpful among respondents who had searched for breast cancer genetics information (n = 283; multiple responses allowed).

Among respondents to the perceived-understanding item (n = 283), 88.7% (251/283) reported some level of understanding of BC genetics, including 18.4% (52/283) who reported a high level of understanding (Fig. 1A). Conversely, 10.2% (29/283) reported poor understanding and 0.4% (1/283) reported no understanding. Medical institutions and physicians were the most cited information source (182/283, 64.3%) and the most frequently rated as most helpful (156/283, 55.1%) (Fig. 1B). Internet sources were also commonly used, including general internet sources (157/283, 55.5%) and hospital websites (138/283, 48.8%).

#### 3.1.3 Perceived participation in treatment decision-making and comprehension difficulties

**Fig. 2:**
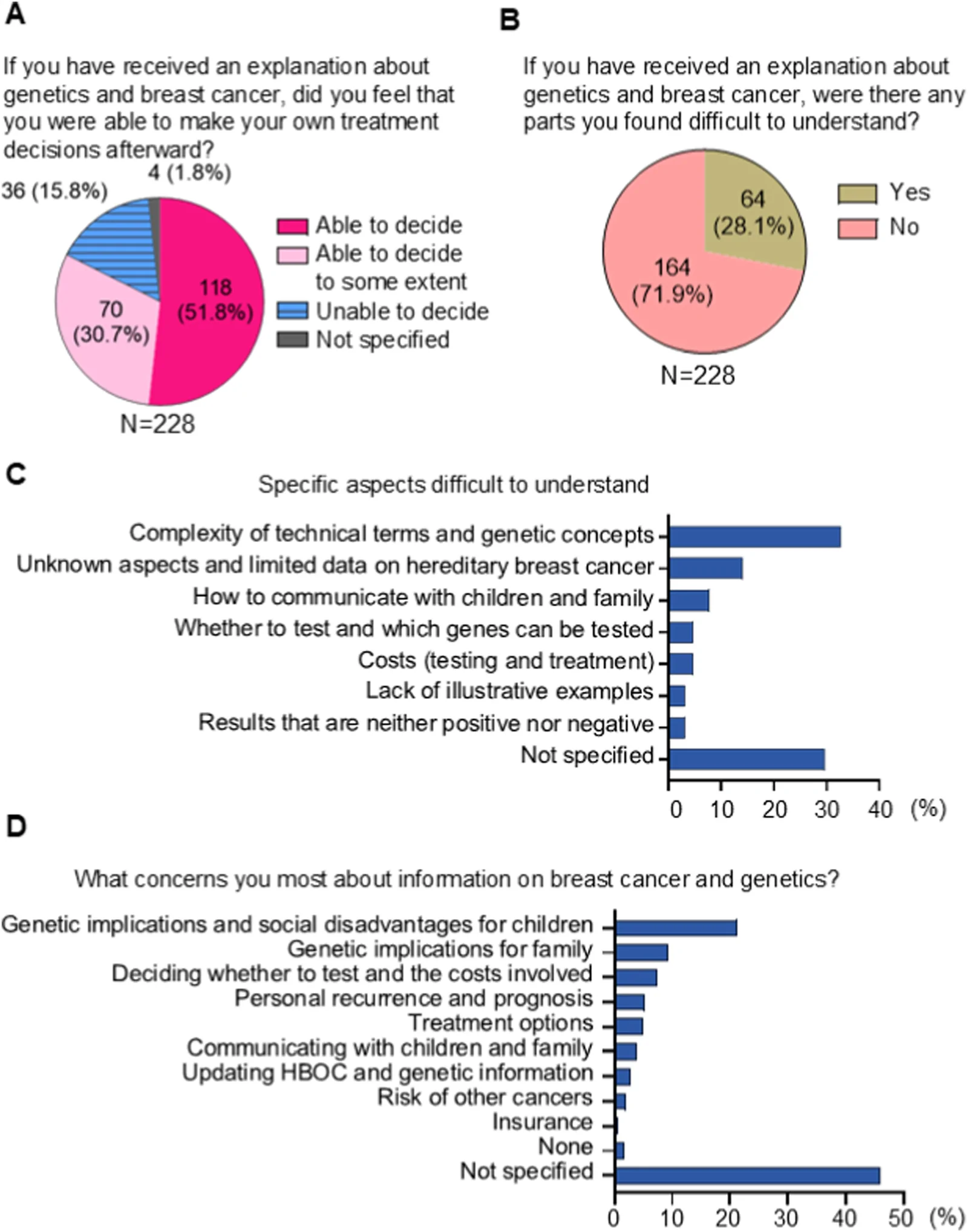
**Perceived participation in treatment decision-making and comprehension barriers**. (A) Perceived ability to make one's own treatment decision after receiving an explanation about breast cancer and genetics (n = 228). (B) Difficulty understanding some aspects among the same respondents (n = 228). (C) Reasons for difficulty among respondents reporting difficulty (n = 64). (D) Main concerns among all patient respondents (n = 375; multiple responses allowed).

**Supplementary Fig. 1:**
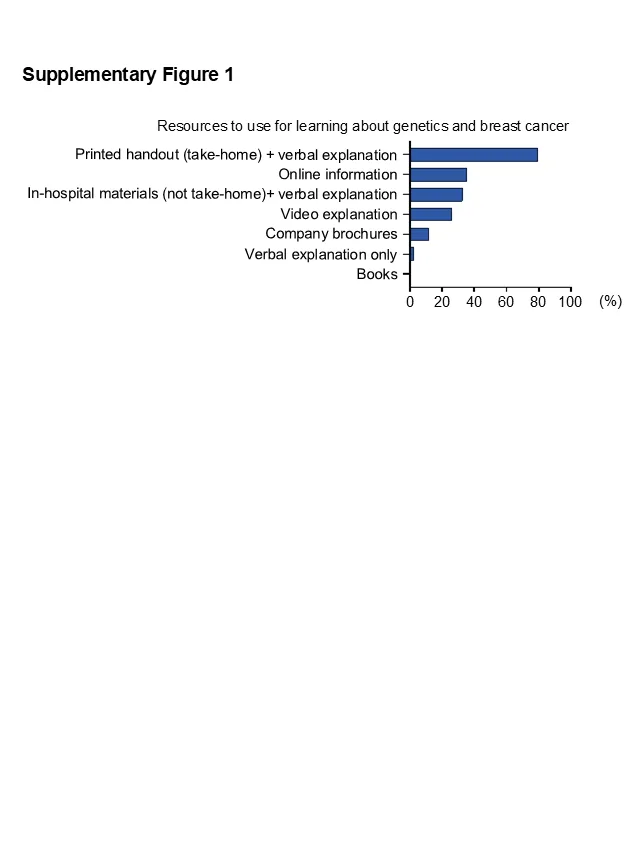
Patient preferences for educational formats (printed materials, oral explanations, online tools) (n = 375; multiple responses allowed).

The decision-making item asked whether, after receiving an explanation about BC and genetics, the respondent felt able to make her own treatment decision. Among respondents to this item (n = 228), 51.8% (118/228) selected “Able to decide”, 30.7% (70/228) selected “Able to decide to some extent”, and 15.8% (36/228) selected “Unable to decide” (Fig. 2A). Among the same 228 respondents, 28.1% (64/228) found that some aspects were difficult to comprehend (Fig. 2B). The most commonly cited specific challenges were the complexity of genetic concepts and medical terminology (21/64, 32.8%), unclear aspects and lack of data on BC and its genetics (9/64, 14.1%), and difficulty explaining the information to children and family members (5/64, 7.8%) (Fig. 2C). Concerns related to BC and its genetics included inheritance by children (80 responses, 21.3%), inheritance by other relatives (35 responses, 9.3%), and associated costs (28 responses, 7.5%) (Fig. 2D). Patient preferences for educational formats are summarized in Supplementary Figure 1.

#### 3.1.4 Patient intentions and preferences for sharing genetic and BC information with family and partners

**Fig. 3:**
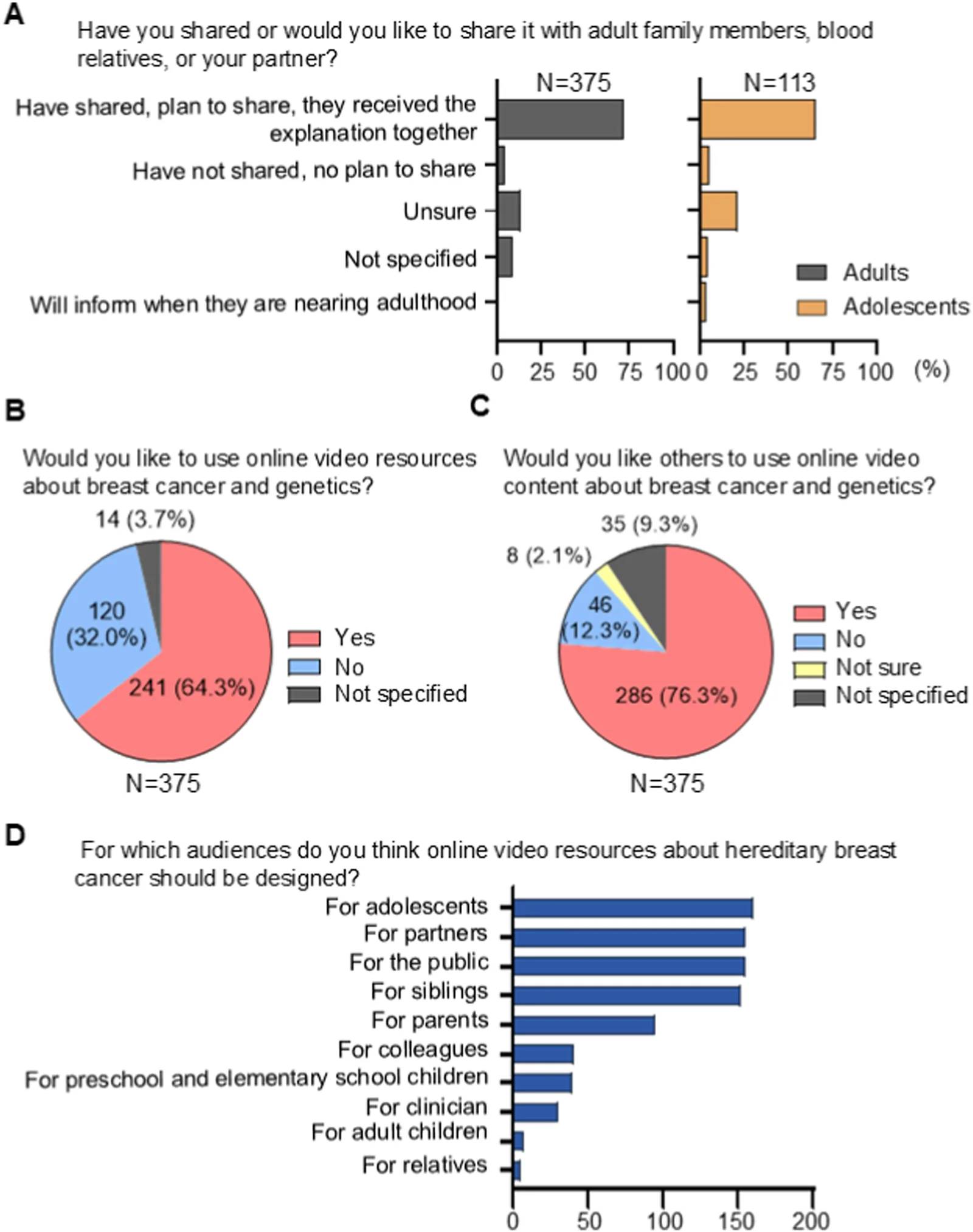
**Family communication intentions and preferences, and stated willingness to use online videos**. (A) Intention to share breast cancer genetics information with adult family members among all patient respondents (n = 375) and with adolescent family members among respondents with at least one adolescent family member (n = 113). (B) Personal willingness to use online video resources (n = 375). (C) Willingness to recommend video resources to others (n = 375). (D) Intended audiences for the video (n = 375; multiple responses allowed).

**Supplementary Fig. 2:**
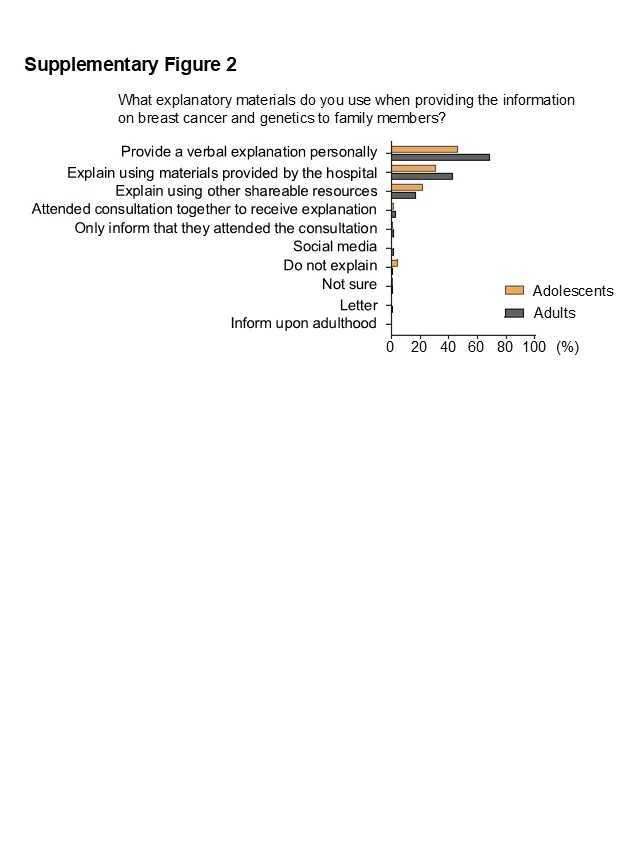
Methods used or intended to share breast cancer genetics information with adult and adolescent family members or relatives among all patient respondents (n = 375 for both items; multiple responses allowed).

Among all respondents (n = 375), 272 (72.5%) expressed a high intention to share or planned to share information about BC and its genetics with their family and partners (Fig. 3A). Among respondents with at least one adolescent family member (n = 113), 74 (65.5%) reported the same intention (Fig. 3A). Co-attending the consultation together with a family member was rarely selected as a preferred method of sharing information, both for adult relatives (10/375, 2.7%) and for adolescent relatives (5/375, 1.3%) (Supplementary Figure 2). Other preferred methods are summarized in Supplementary Fig. 2. Regarding online video resources, 241/375 (64.3%) respondents indicated they would themselves like to use such resources for information about BC genetics (Fig. 3B); a higher proportion (286/375, 76.3%) indicated they would like others to use these resources (Fig. 3C). Intended audiences for the videos are shown in Fig. 3D.

### 3.2 Healthcare provider survey

#### 3.2.1 Characteristics of healthcare provider respondents

Table 2 shows the characteristics of the healthcare provider respondents. Thirty providers answered the provider survey items analyzed in this study. Of these, 16 were affiliated with an HBOC core facility, 2 with an HBOC cooperative facility, and 10 with facilities not falling into either category. Two had an unknown affiliation. Among those at HBOC core facilities, 11 (68.8%) were located in Kanto including Tokyo, with cancer centers and university hospitals representing 8 (50.0%) and 7 (43.8%) of the high-volume centers, respectively.

**Table 2:** Characteristics of healthcare provider respondents.

N	30	HBOC Core facility	HBOC cooperative facility	Other/None of the above	Unknown
		16 (53.3)	2 (6.7)	10 (33.3)	2 (6.7)
Region	Kanto	11 (68.8)	0 (0)	6 (60)	1 (50)
	Chubu	2 (12.5)	0 (0)	0 (0)	0 (0)
	Kinki	2 (12.5)	0 (0)	3 (30)	0 (0)
	Chugoku/Shikoku	1 (6.3)	1 (50)	0 (0)	1 (50)
	Kyushu	0 (0)	1 (50)	1 (10)	0 (0)
Facility type	Cancer Center	8 (50)	0 (0)	1 (10)	0 (0)
	Clinic	1 (6.3)	0 (0)	2 (20)	0 (0)
	Univ. hosp.	7 (43.8)	0 (0)	1 (10)	1 (50)
	Municipal hosp.	0 (0)	2 (100)	6 (60)	1 (50)
Professional role	Dr (Breast Surgeon)	12 (75)	1 (50)	3 (30)	1 (50)
	Dr. (Oncologist)	2 (12.5)	0 (0)	0 (0)	0 (0)
	Dr. (Gynecologist, others)	0 (0)	0 (0)	3 (30)	0 (0)
	Nurse	1 (6.3)	1 (50)	1 (10)	1 (50)
	Laboratory Technician	0 (0)	0 (0)	3 (30)	0 (0)
	Physical Therapist	1 (6.3)	0 (0)	0 (0)	0 (0)
Genetic counseling department	Yes	16 (100)	0 (0)	3 (30)	2 (100)
	No	0 (0)	1 (50)	6 (60)	0 (0)
	Unknown	0 (0)	1 (50)	1 (10)	0 (0)
Certified genetic counselor	Yes	14 (87.5)	0 (0)	4 (40)	1 (50)
	No	2 (12.5)	2 (100)	5 (50)	1 (50)
	Unknown	0 (0)	0 (0)	1 (10)	0 (0)
Opportunities to contact patients or clients	Yes	14 (87.5)	2 (100)	6 (60)	2 (100)
	No	2 (12.5)	0 (0)	4 (40)	0 (0)

#### 3.2.2 Practices and challenges in providing information on BC and its genetics in clinical settings

**Fig. 4:**
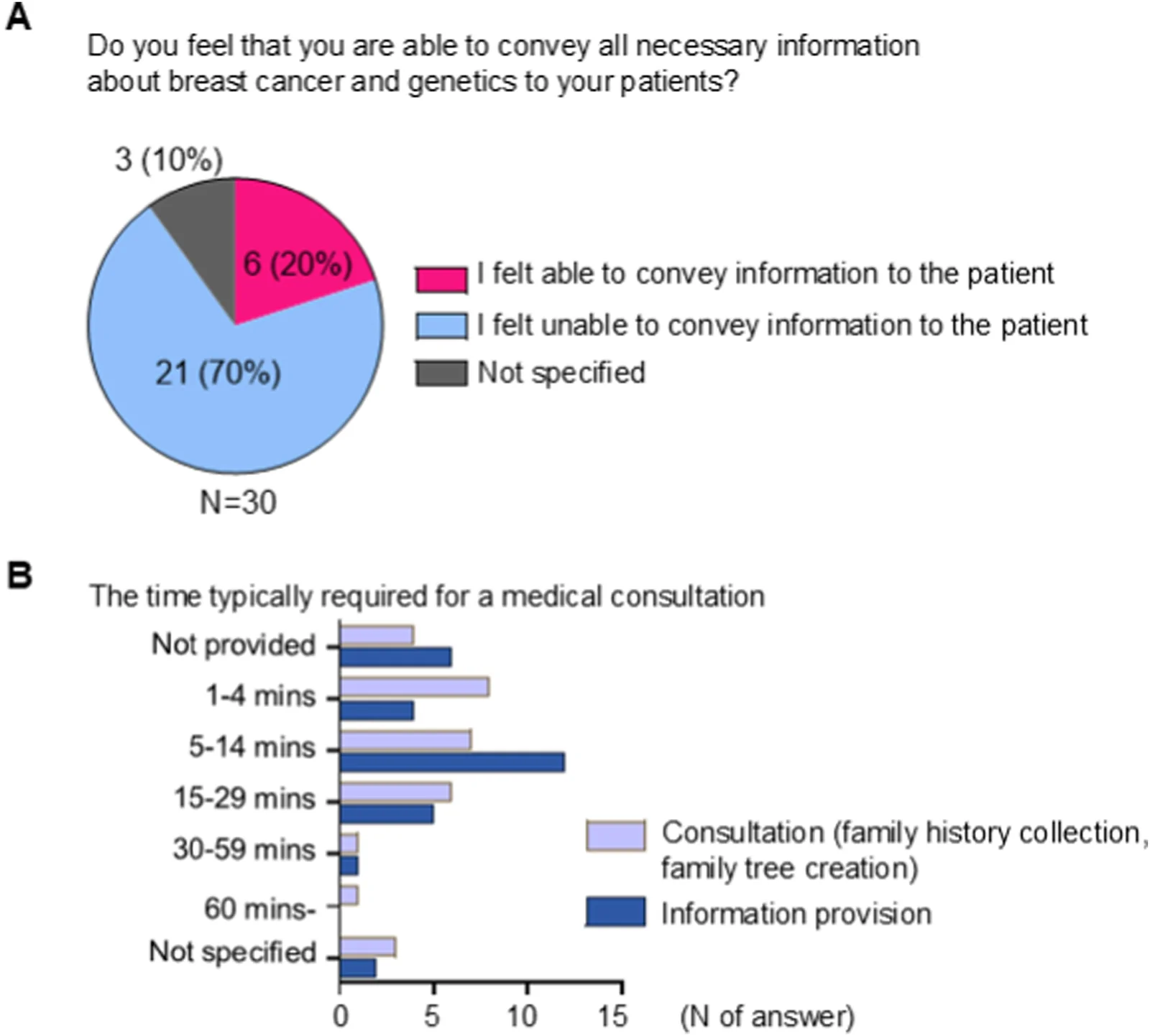
**Providers' practices and perceived constraints in communicating breast cancer genetics information**. (A) Perceived ability to convey all necessary information in routine practice (n = 30). (B) Typical time spent on pedigree creation and on providing genetics-related information (n = 30).

**Supplementary Fig. 3:**
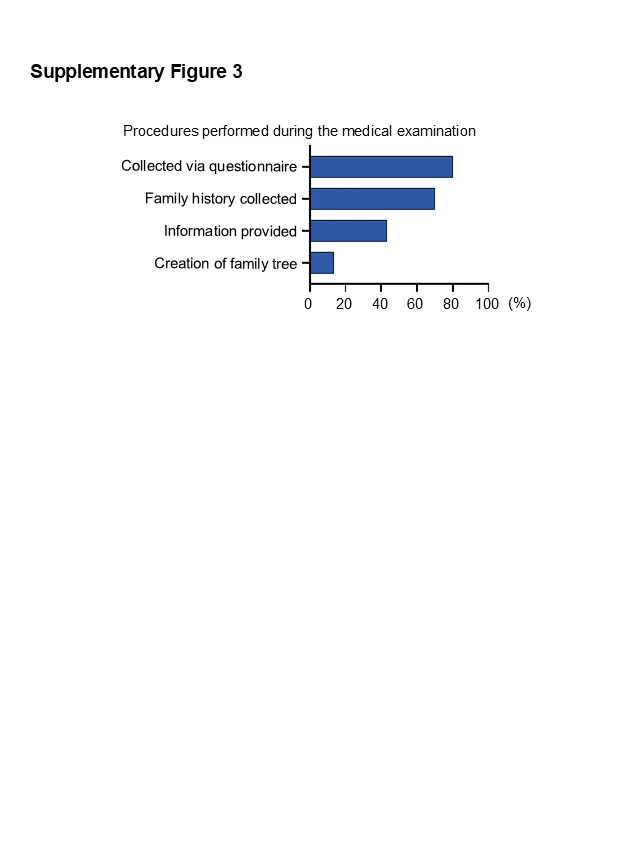
Common procedures at initial consultation when hereditary breast cancer is suspected (n = 30; multiple responses allowed).

**Supplementary Fig. 4:**
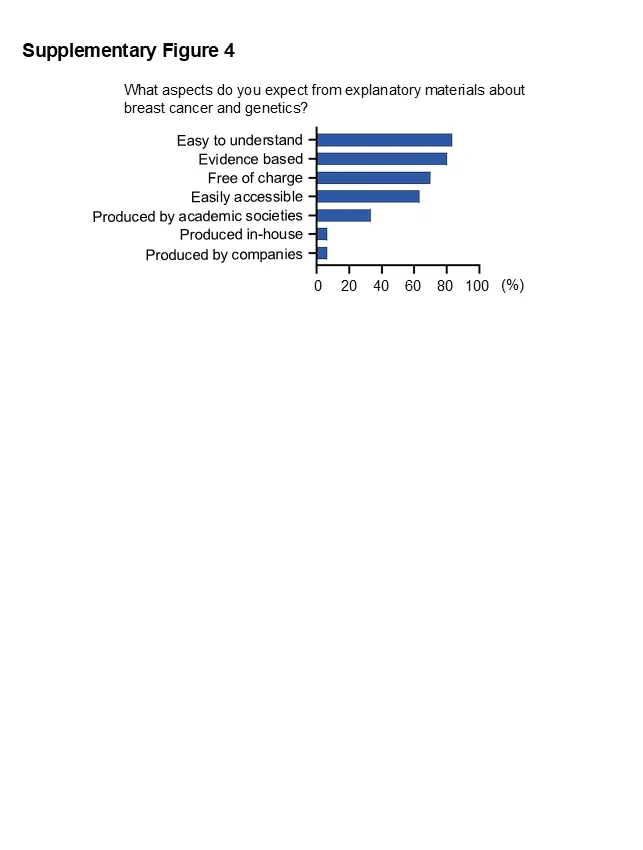
Characteristics of educational materials prioritized by providers (n = 30).

Fig. 4A shows providers’ perceived ability to convey information to patients: 6/30 (20.0%) felt able to convey all necessary information, whereas 21/30 (70.0%) did not feel able. The questionnaire did not separately assess whether this perceived inability reflected gaps in training or expertise, time constraints, or both. When hereditary BC was suspected at the first consultation, the most common procedures included taking patient history (24/30, 80.0%), collecting family history (21/30, 70.0%), and providing information (13/30, 43.3%) (Supplementary Fig. 3). The time required for the initial information provision varied, with 1–4 min most common for collecting family history and pedigree creation (8/30, 26.7%) and 5–14 min most frequent for providing information (12/30, 40.0%) (Fig. 4B). Desired characteristics of explanatory media were clarity (83.3%) and evidence-based content (80.0%) (Supplementary Fig. 4).

#### 3.2.3 Challenges in providing explanation about BC and its genetics and the use of online resources

**Fig. 5:**
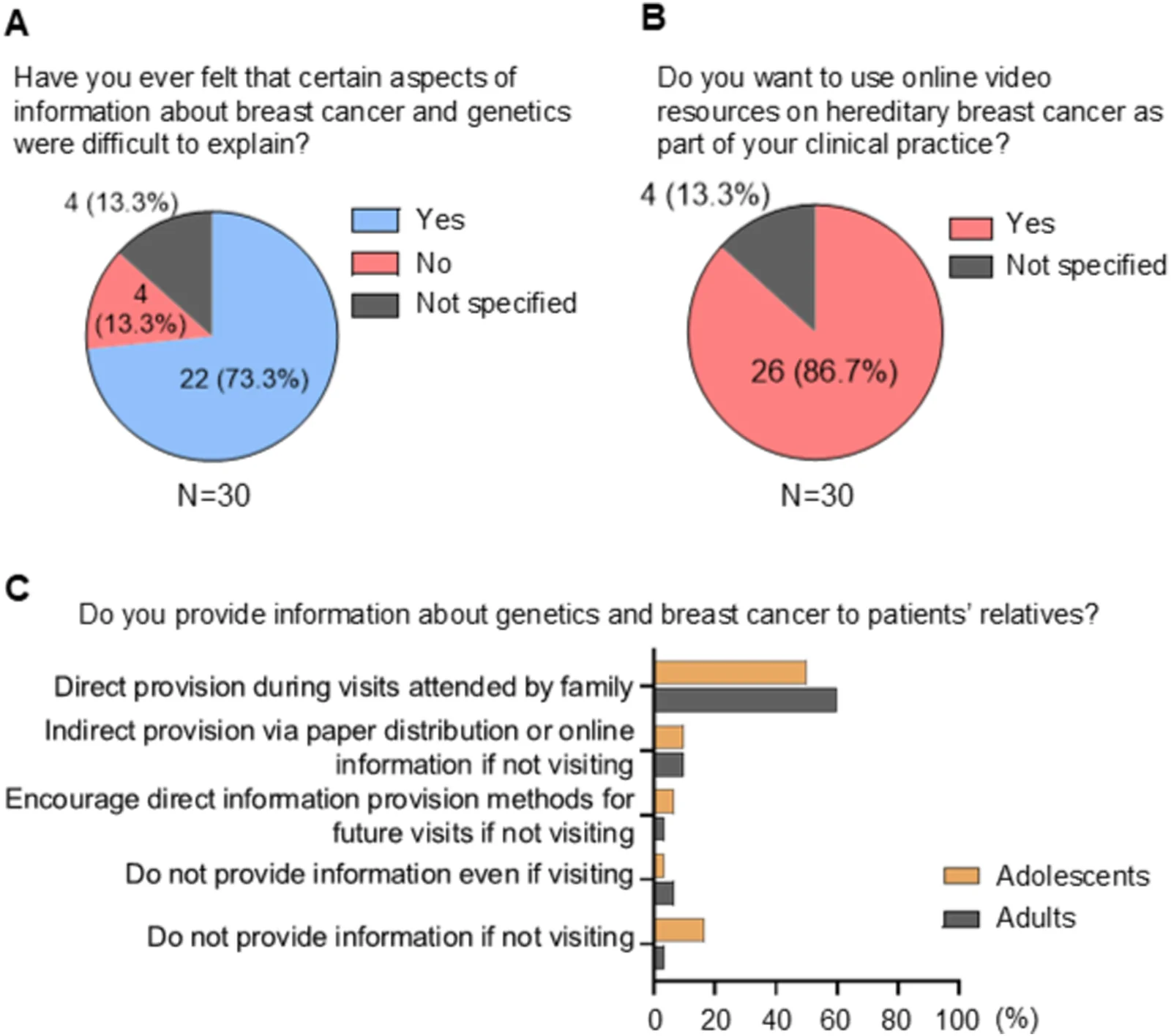
**Provider-reported communication challenges and intended use of online videos**. (A) Whether providers experience difficulty explaining breast cancer genetics (n = 30). (B) Stated willingness to use online video resources in clinical practice (n = 30). (C) Usual methods for providing information to adult family members and to adolescents (n = 30).

**Supplementary Fig. 5:**
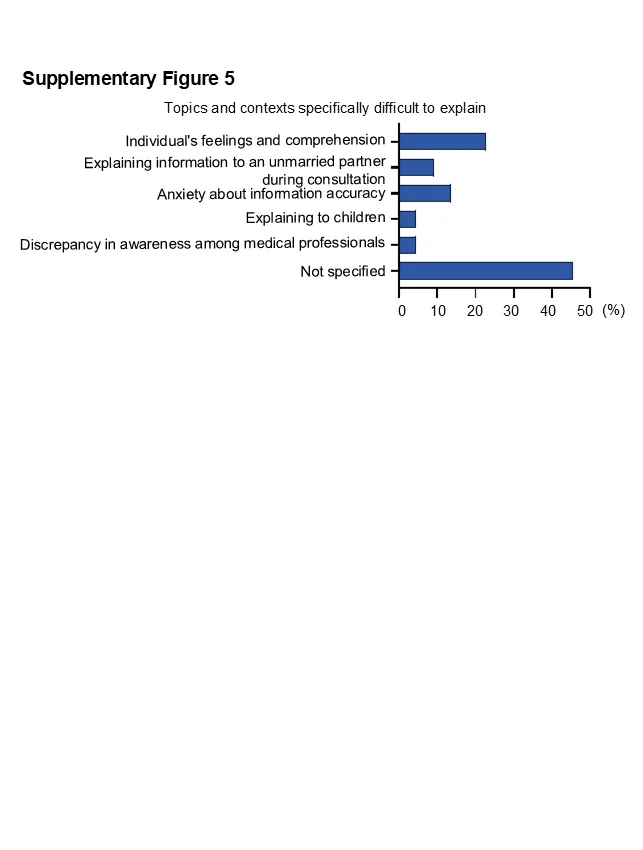
Topics/contexts reported as difficult to explain (free-text responses; coded post hoc) (n = 22 providers contributing free-text responses).

Among respondents to this item (n = 30), 22 (73.3%) found it challenging to explain information related to BC and its genetics (Fig. 5A). Interest in using online video information to assist clinical operations was high: 26/30 (86.7%) respondents expressed a stated willingness to use such resources, and none opposed their use (Fig. 5B). Common themes from the open-ended responses included individuals' feelings and comprehension (22.7%), anxiety about information accuracy (13.6%), and explaining information to an unmarried partner during consultation (9.1%) (Supplementary Fig. 5). When providing genetic and BC information to patients’ families, 18/30 (60%) respondents would directly provide details during consultations attended by adult family members; if the family did not attend, indirect methods such as distribution of paperwork and provision of online information were used by 3/30 (10%). For adolescents, direct provision during consultations attended by adult family members occurred in 50% (15/30) of cases, with indirect methods used in 10% (3/30). Notably, 16.7% (5/30) of providers did not provide information at all to adolescents who did not visit, a higher rate than for adults (Fig. 5C).

### 3.3 Comparison of patient and provider perspectives

**Supplementary Fig. 6:**
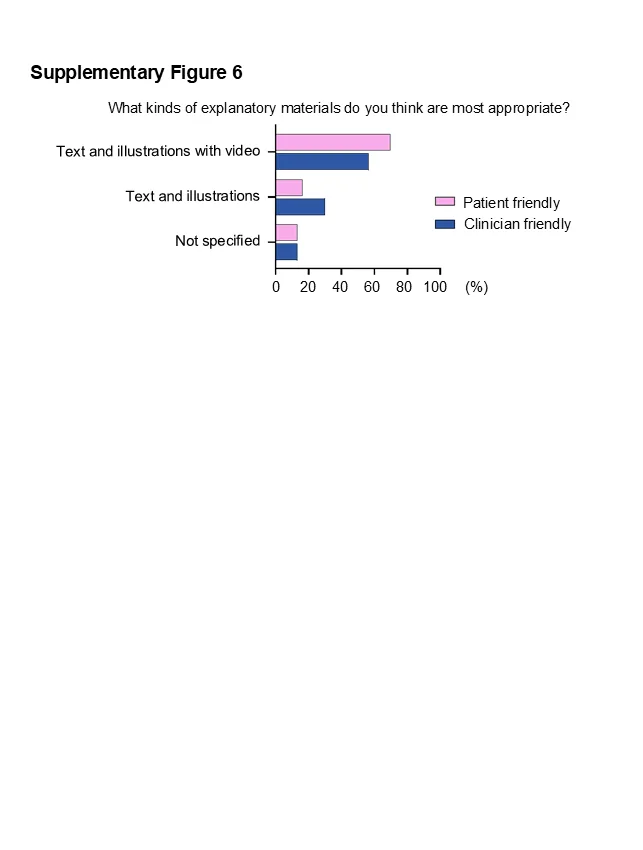
Perceived user-friendly formats of explanatory materials (patients vs providers) (patients: n = 375; providers: n = 30).

Direct comparison between the patient and provider samples is limited by the seven-month gap between the two data-collection periods, the different recruitment contexts, and the likely differences in respondent characteristics. With this caveat, providers more often selected videos combining text and illustrations as a user-friendly format for explaining information about BC and genetics: 56.7% (17/30) selected this format as most user-friendly, and 70.0% (21/30) indicated that it would be easy for patients to use. In contrast, 16.7% of patients selected text-and-illustration formats as user-friendly (Supplementary Fig. 6). Overall, both samples suggested a preference for evidence-based content presented in visually accessible formats.

## 4 Discussion

This descriptive cross-sectional survey suggests that, among patients and providers already engaged with digital health information and educational resources, evidence-based online videos are viewed as a potentially useful adjunct to hereditary BC communication in Japan. However, participants were not exposed to a specific video intervention; therefore, the study assessed perceived usefulness and stated willingness to use online video resources rather than demonstrated acceptability or effectiveness of an implemented tool. [18]

### 4.1 Information sources, comprehension, and digital health information

Patients relied strongly on physicians and medical institutions while also using internet sources. Although most respondents reported some understanding of BC genetics, a substantial minority reported comprehension difficulties, consistent with the complexity of hereditary risk information and the role of counseling and decision aids in supporting informed decision-making. [6,7] These findings also reflect the broader “quality versus reach” challenge in online hereditary BC information. [16] Linking trusted resources to clinical care, such as through QR codes, hospital websites, or patient portals, may improve access to reliable information, but these strategies require prospective evaluation. Tools such as DISCERN, PEMAT, and eHEALS may also help evaluate future digital materials. [[19], [20], [21]]

### 4.2 Provider perspectives and time constraints

Most providers reported difficulty conveying all necessary information in routine practice, although the questionnaire did not distinguish whether this reflected training gaps, time constraints, or both. Evidence-based videos may help standardize core explanations and allow review outside the consultation, consistent with prior decision-aid literature, [6,7] but this should be evaluated prospectively. Larger studies should also examine differences across provider roles, because the present sample was too small and heterogeneous for role-stratified analysis.

### 4.3 Family communication

Intentions to share genetic information with adult relatives were common, and many respondents with adolescent family members also intended to share information with them. Although evidence specific to *BRCA1/2* disclosure remains limited in Japan, broader literature on hereditary cancer and genetic-result disclosure shows that intra-family communication is variable and that probands often act as information gatekeepers within families. [22,23] Communication with at-risk minors and adolescents is particularly complex because it requires developmentally appropriate information and support for parents. Japanese cultural and structural factors, including the relatively recent expansion of insured genetic services and limited public-facing materials for adolescents, may further shape these communication patterns. Our findings support future evaluation of age-appropriate, family-centered educational resources, including shareable video materials for patients and relatives.

### 4.4 Strengths and limitations

This study includes both patient and provider perspectives, addresses a topic with limited published Japanese data, and reports item-specific denominators throughout. However, selection bias is substantial: the patient response rate was approximately 2.2% (375/17,076), and providers were recruited from a non-enumerable convenience sample at a video-education booth. Respondents were therefore likely more receptive to digital educational tools than broader patient or provider populations.

The patient survey did not collect individual cancer diagnosis or genetic-testing status, limiting the direct applicability of the findings to women with confirmed HBOC or those undergoing genetic counseling. The provider sample was small and heterogeneous, preventing meaningful role-stratified analyses, and the patient and provider surveys differed in timing and recruitment context.

The questionnaires were not formally validated, and several terms were not pre-defined for respondents. Provider free-text responses were coded descriptively without a formal coding guide or inter-rater reliability statistic. All findings are self-reported; no validated knowledge or decisional-conflict instruments were used, and no inferential statistical tests were performed. Finally, several authors are affiliated with BC TUBE or Peer Ring, and these non-financial interests should be considered when interpreting the findings.

### 4.5 Conclusions

In two selected samples, patients and providers supported evidence-based online videos as an adjunct to hereditary BC communication in Japan. Family communication about hereditary risk was common but not universal. Because respondents were self-selected and already engaged with digital health information or educational resources, the findings should not be generalized to broader Japanese patient or provider populations.

### 4.6 Potential practice implications and future research

Evidence-based online videos may help standardize core information, support review outside the consultation, and facilitate family communication, including with adolescent family members. These remain hypotheses for prospective evaluation. Future studies should expose participants to defined video interventions, use validated outcome measures such as knowledge, decisional conflict, anxiety, satisfaction, and family communication, include more representative samples, and assess differences across provider roles before firm implementation recommendations are made.

## Ethical statement

This study was reviewed and approved by the Ethical Review Board of Ichinomiya Nishi Hospital (Approval No. 2022002). All procedures involving human participants were performed in accordance with the ethical standards of the institutional and/or national research committee and with the 1964 Helsinki Declaration and its later amendments, as well as applicable Japanese ethical guidelines for medical research. In the recruitment email, potential participants were informed of the study objectives, investigator contact details, assurance of anonymity, and their right to decline or withdraw at any time. On the introductory page of the Google Form questionnaire, it was explicitly stated that beginning the survey implied informed consent to participate.

## Authors' contributions

Conceptualization: Asumi Iesato; Data curation: Asumi Iesato; Funding acquisition: Asumi Iesato; Methodology: Asumi Iesato, Hitomi Suzuki, Rie Tahara, Mitsuo Terada, Yuko Nakazawa, Madoka Iwase, Tomoko Shibayama, Chihiro Kawamura, Nami Yamashita, Atsushi Fushimi; Investigation: Asumi Iesato, Hitomi Suzuki; Writing – original draft: Asumi Iesato; Writing – review and editing: Hitomi Suzuki, Rie Tahara, Mitsuo Terada, Yuko Nakazawa, Madoka Iwase, Tomoko Shibayama, Chihiro Kawamura, Nami Yamashita, Atsushi Fushimi.

## Availability of data and materials

To protect participant confidentiality, raw questionnaire response files were not submitted, as open access to such data could potentially lead to the identification of individuals. The datasets used and analyzed during the current study are available from the corresponding author on reasonable request.

## Funding

This research was funded by Project Mirai Cancer Research Grants from Relay for Life, 10.13039/100019727Japan Cancer Society (Tokyo, Japan) to Asumi Iesato.

## Declaration of competing interests

The authors declare that they have no known competing financial interests or personal relationships that could have appeared to influence the work reported in this paper.
