# Supplementary material for: Perceived usefulness of online videos for hereditary breast cancer communication in Japan: Patient and provider perspectives
Source: Public Health Pract (Oxf). 2026 Aug 22;12:100845. doi: 10.1016/j.puhip.2026.100845 (PMC13543082; doi:10.1016/j.puhip.2026.100845)
Supplement: Supplementary file 1 [file mmc4.docx]

STROBE Statement—checklist of items that should be included in reports of observational studies

|  | Item No. | Recommendation | Page  No. | Relevant text from manuscript |
| --- | --- | --- | --- | --- |
| **Title and abstract** | 1 | (*a*) Indicate the study’s design with a commonly used term in the title or the abstract | 2 | We conducted a cross-sectional web-based survey of female patients registered with a Japanese online peer-support community for women diagnosed with breast, cervical, or ovarian cancers (Peer Ring; January–February 2024) and healthcare providers recruited at the Japanese Breast Cancer Society Annual Meeting (June 29–July 1, 2023). |
|  |  | (*b*) Provide in the abstract an informative and balanced summary of what was done and what was found | 2 | ・We aimed to describe patterns of information seeking and provision and to evaluate the acceptability and perceived usefulness of evidence-based online video resources for hereditary breast cancer communication in Japan.  ・We conducted a cross-sectional web-based survey of female patients registered with a Japanese peer-support community for women diagnosed with breast, cervical, or ovarian cancers and healthcare providers involved in breast cancer care.  ・Most patients relied on information from clinicians and medical institutions, although a substantial minority reported gaps in understanding. While most patients intended to share genetic information with family members, only a small proportion of healthcare providers felt able to convey all necessary information within routine practice. Most providers expressed interest in using evidence-based online video resources.  ・Evidence-based online videos were perceived as an acceptable adjunct to hereditary breast cancer communication and may support patient understanding, family communication, and efficient use of limited consultation time. |
| Introduction | | | |  |
| Background/rationale | 2 | Explain the scientific background and rationale for the investigation being reported | 3 | Effective patient education and clinical communication are essential for preference-sensitive decisions in hereditary cancer care. However, hereditary risk information is cognitively demanding, and limited consultation time can constrain opportunities to ensure comprehension, address emotions, and support family communication. Scalable educational resources that patients can revisit outside the clinic may therefore complement in-person counseling and help align understanding across patients and family members. |
| Objectives | 3 | State specific objectives, including any prespecified hypotheses | 3 | Therefore, we surveyed members of an online female cancer patient community and healthcare providers to describe patterns of information seeking/provision and to evaluate the acceptability and perceived usefulness of evidence-based online video resources. |
| Methods | | | |  |
| Study design | 4 | Present key elements of study design early in the paper | 2 | We conducted a cross-sectional web-based survey of female patients registered with a Japanese online peer-support community for women diagnosed with breast, cervical, or ovarian cancers (Peer Ring; January–February 2024) and healthcare providers recruited at the Japanese Breast Cancer Society Annual Meeting (June 29–July 1, 2023). |
| Setting | 5 | Describe the setting, locations, and relevant dates, including periods of recruitment, exposure, follow-up, and data collection | 3,4 | ・We invited all registered members of the online peer-support community “Peer Ring”— a Japan-based platform serving women diagnosed with breast, cervical, and ovarian cancers—to participate in our survey from January to February 2024.  ・At the 2023 Japanese Breast Cancer Society meeting (June 29–July 1), we invited booth visitors who self-identified as healthcare professionals routinely involved in breast cancer care to participate in the survey. |
| Participants | 6 | (*a*) *Cohort study*—Give the eligibility criteria, and the sources and methods of selection of participants. Describe methods of follow-up  *Case-control study*—Give the eligibility criteria, and the sources and methods of case ascertainment and control selection. Give the rationale for the choice of cases and controls  *Cross-sectional study*—Give the eligibility criteria, and the sources and methods of selection of participants | 3,4 | ・We invited all registered members of the online peer-support community “Peer Ring”— a Japan-based platform serving women diagnosed with breast, cervical, and ovarian cancers—to participate in our survey from January to February 2024.  ・Participation was not limited by cancer type, and we did not include an item asking respondents to specify their diagnosis.  At the 2023 Japanese Breast Cancer Society meeting (June 29–July 1), we invited booth visitors who self-identified as healthcare professionals routinely involved in BC care (e.g., medical oncologists, breast surgeons, genetic counselors, and oncology nurses); eligibility was confirmed through a brief oral screening. |
|  |  | (*b*) *Cohort study*—For matched studies, give matching criteria and number of exposed and unexposed  *Case-control study*—For matched studies, give matching criteria and the number of controls per case |  | Not applicable. |
| Variables | 7 | Clearly define all outcomes, exposures, predictors, potential confounders, and effect modifiers. Give diagnostic criteria, if applicable | 2 | Items addressed information sources and perceived understanding of breast cancer genetics, perceived influence on treatment decision-making, intentions to share information within families, preferred formats for educational materials, and willingness to use evidence-based online videos in clinical practice. |
| Data sources/ measurement | 8* | For each variable of interest, give sources of data and details of methods of assessment (measurement). Describe comparability of assessment methods if there is more than one group | 3,4 | ・The questionnaire was developed in consultation with the BC TUBE editorial committee and the Peer Ring operations team  ・It was then administered via Google Forms.  ・The questionnaire was administered in Japanese.  ・In total, 32 questions were included in the survey.  ・In total, 27 questions were included in the survey. |
| Bias | 9 | Describe any efforts to address potential sources of bias | 8 | ・Response rates were low and selection bias is possible, particularly among providers recruited at a video-education booth.  ・The study relied on self-report. |
| Study size | 10 | Explain how the study size was arrived at | 3 | ・As this study was exploratory, we distributed the questionnaire to all Peer Ring members to collect as many responses as possible.  ・Although no formal *a priori* sample size calculation was performed, a prior internal survey by the BC TUBE editorial committee and Peer Ring operations team obtained 360 patient responses and yielded meaningful insights. |

Continued on next page

| Quantitative variables | 11 | Explain how quantitative variables were handled in the analyses. If applicable, describe which groupings were chosen and why | 2,4 | ・Analyses were descriptive.  ・Graphs were generated using GraphPad Prism 8; no statistical tests were performed.  ・Free-text responses were qualitatively coded using an inductive approach, and categories were summarized as counts. |
| --- | --- | --- | --- | --- |
| Statistical methods | 12 | (*a*) Describe all statistical methods, including those used to control for confounding | 2,4 | ・Analyses were descriptive.  ・No statistical tests were performed.  ・No inferential statistics or interrater reliability testing were performed. |
|  |  | (*b*) Describe any methods used to examine subgroups and interactions |  | Not applicable. |
|  |  | (*c*) Explain how missing data were addressed |  | Not applicable. |
|  |  | (*d*) *Cohort study*—If applicable, explain how loss to follow-up was addressed  *Case-control study*—If applicable, explain how matching of cases and controls was addressed  *Cross-sectional study*—If applicable, describe analytical methods taking account of sampling strategy |  | Not applicable. |
|  |  | (*e*) Describe any sensitivity analyses |  | Not applicable. |
| Results | | | | |
| Participants | 13* | (a) Report numbers of individuals at each stage of study—eg numbers potentially eligible, examined for eligibility, confirmed eligible, included in the study, completing follow-up, and analysed | 4,6 | ・Of the 17,076 invited Peer Ring members, 375 completed the patient survey (response rate: approximately 2.2%).  ・Table 2 shows the characteristics of the 29 healthcare providers who completed the survey. |
|  |  | (b) Give reasons for non-participation at each stage |  | Not applicable. |
|  |  | (c) Consider use of a flow diagram |  | Not applicable. |
| Descriptive data | 14* | (a) Give characteristics of study participants (eg demographic, clinical, social) and information on exposures and potential confounders | 4,5,6 | ・Participants were categorized by genetic counseling status and information-seeking behavior into three primary strata.  ・In terms of age, respondents in their 50s were most frequently represented.  ・Regarding family structure, the proportions reporting a partner/spouse were 77.4% (NC/NS), 75.1% (NC/S), and 78.2% (C/S); the proportions reporting siblings were 86.0%, 92.7%, and 88.5%, respectively.  ・Table 1  ・Table 2 shows the characteristics of the 29 healthcare providers who completed the survey |
|  |  | (b) Indicate number of participants with missing data for each variable of interest | 2,5,6 | ・Among 375 patient respondents, 88.7% reported some level of understanding of breast cancer genetics (283 respondents to this item), while 28.1% reported difficulty understanding some aspects (228 respondents).  ・In a survey of 283 respondents, 52 (18.4%) reported a high level of perceived understanding of information regarding BC and genetics.  ・A survey of 228 respondents revealed the impact of information on BC and its genetics on treatment decision-making.  ・Among those with at least one adolescent family member (n = 113), 74 (65.4%) reported the same intention |
|  |  | (c) *Cohort study*—Summarise follow-up time (eg, average and total amount) |  | Not applicable. |
| Outcome data | 15* | *Cohort study*—Report numbers of outcome events or summary measures over time |  | Not applicable. |
|  |  | *Case-control study—*Report numbers in each exposure category, or summary measures of exposure |  | Not applicable. |
|  |  | *Cross-sectional study—*Report numbers of outcome events or summary measures | 2 | ・Among 375 patient respondents, 88.7% reported some level of understanding of breast cancer genetics (283 respondents to this item), while 28.1% reported difficulty understanding some aspects (228 respondents).  ・Among 29 providers, only 20% felt able to convey all necessary information in routine practice, and 86.7% expressed interest in using online video resources |
| Main results | 16 | (*a*) Give unadjusted estimates and, if applicable, confounder-adjusted estimates and their precision (eg, 95% confidence interval). Make clear which confounders were adjusted for and why they were included |  | Not applicable.  This study was descriptive and did not estimate associations or adjusted effects. |
|  |  | (*b*) Report category boundaries when continuous variables were categorized | 6 | The time required for the initial information provision varied, with 1–4 min being the most common for collecting family history and pedigree creation (n = 8, 26.7%). In contrast, a duration of 5–14 min was most frequently allocated for providing information (n = 12, 40%) |
|  |  | (*c*) If relevant, consider translating estimates of relative risk into absolute risk for a meaningful time period |  | Not applicable.  Relative risk estimates were not calculated in this cross-sectional descriptive study. |

Continued on next page

| Other analyses | 17 | Report other analyses done—eg analyses of subgroups and interactions, and sensitivity analyses | 4 | ・Participants were categorized by genetic counseling status and information-seeking behavior into three primary strata: no genetic counseling/no genetic information search (NC/NS) (n = 93, 24.8%), no genetic counseling/genetic information search (NC/S) (n = 193, 51.5%), and genetic counseling/genetic information search (C/S) (n = 87, 23.2%)  ・The provider survey included an open-ended item asking respondents to describe topics or contexts they found difficult to explain about BC and genetics. Free-text responses were qualitatively coded using an inductive approach, and categories were summarized as counts. |
| --- | --- | --- | --- | --- |
| Discussion | | | | |
| Key results | 18 | Summarise key results with reference to study objectives | 8 | This survey describes how patients and families obtain and use information about hereditary breast cancer and how healthcare providers currently provide such information. |
| Limitations | 19 | Discuss limitations of the study, taking into account sources of potential bias or imprecision. Discuss both direction and magnitude of any potential bias | 8 | Several limitations should be noted. Response rates were low and selection bias is possible, particularly among providers recruited at a video-education booth. The study relied on self-report and did not capture genetic test status or objective outcomes using validated instruments |
| Interpretation | 20 | Give a cautious overall interpretation of results considering objectives, limitations, multiplicity of analyses, results from similar studies, and other relevant evidence | 8 | ・Accordingly, these results should be interpreted as descriptive and hypothesis-generating, intended to inform implementation planning rather than support inferential claims.  ・For context, a study from the USA reported that 52% of participants who received results from a genetics provider shared them with all first-degree relatives |
| Generalisability | 21 | Discuss the generalisability (external validity) of the study results | 8 | Response rates were low and selection bias is possible, particularly among providers recruited at a video-education booth |
| Other information | |  | | |
| Funding | 22 | Give the source of funding and the role of the funders for the present study and, if applicable, for the original study on which the present article is based | 9 | This research was funded by Project Mirai Cancer Research Grants from Relay for Life, Japan Cancer Society |

*Give information separately for cases and controls in case-control studies and, if applicable, for exposed and unexposed groups in cohort and cross-sectional studies.

**Note:** An Explanation and Elaboration article discusses each checklist item and gives methodological background and published examples of transparent reporting. The STROBE checklist is best used in conjunction with this article (freely available on the Web sites of PLoS Medicine at http://www.plosmedicine.org/, Annals of Internal Medicine at http://www.annals.org/, and Epidemiology at http://www.epidem.com/). Information on the STROBE Initiative is available at www.strobe-statement.org.
